# Perceptions of harm from secondhand smoke exposure among U.S. adults, 2009–2010

**DOI:** 10.1186/s12971-016-0069-8

**Published:** 2016-02-02

**Authors:** Judy Kruger, Roshni Patel, Michelle Kegler, Steven D. Babb, Brian A. King

**Affiliations:** Office on Smoking and Health, National Center for Chronic Disease Prevention and Health Promotion, Centers for Disease Control and Prevention, Atlanta, GA USA; Contractor Support for NCCDPHP/NGIS, Office on Smoking and Health, National Center for Chronic Disease Prevention and Health Promotion, Atlanta, GA USA; Emory University, Rollins School of Public Health, Atlanta, GA USA

**Keywords:** Tobacco, Smoking, Policy, Secondhand smoke

## Abstract

**Background:**

Exposure to secondhand smoke (SHS) causes significant disease and death. We assessed the prevalence and correlates of perceptions about the health harm of SHS among U.S. adults at the national and state level.

**Methods:**

Data came from the 2009–2010 National Adult Tobacco Survey, a national landline and cellular telephone survey. Perceptions about the health harms of SHS were assessed as follows: ‘not at all harmful’, ‘somewhat harmful’, and ‘very harmful’. Descriptive statistics were used to assess the prevalence of SHS harm perceptions by tobacco use and sociodemographic factors, including sex, age, race/ethnicity, education, marital status, annual household income, region, sexual orientation, children in the household, and smoke-free law coverage. Logistic regression was used to assess odds of perceiving SHS to be “very harmful” (vs. “not at all harmful” or “somewhat harmful”), adjusting for the aforementioned factors.

**Results:**

Nationally, 64.5 % of adults perceived SHS as ‘very harmful’ (state range: 73.5 % [Utah] to 53.7 % [Kentucky]). By tobacco use, the perception that SHS is ‘very harmful’ was: 76.5 % among nonusers of tobacco; 62.1 % among noncombustible only users; 47.9 % among combustible only users; and 40.8 % among dual combustible and noncombustible users. Following adjustment, the perception that SHS was ‘very harmful’ was higher among females, non-Hispanic minorities and Hispanics, respondents living with children, and states with 100 % smoke-free law coverage. Among current tobacco users the odds of perceiving SHS to be ‘very harmful’ was lower in the Midwest than the West.

**Conclusions:**

Almost two-thirds of American adults perceive SHS as ‘very harmful’; however, currently only half of all Americans are protected by comprehensive state or local smoke-free laws. These findings underscore the importance of public education campaigns to increase awareness of SHS exposure harm and the benefits of smoke-free environments. Expanding comprehensive smoke-free laws could protect all Americans from SHS.

## Background

Secondhand smoke (SHS) exposure causes heart disease, stroke, and lung cancer in nonsmoking adults, and sudden infant death syndrome, acute respiratory infections, middle ear infections, and more severe asthma in children [1]. SHS exposure causes more than 41,000 deaths among U.S. nonsmokers each year, costing the country an estimated $5.6 billion annually [2]. The U.S. Surgeon General has concluded that there is no risk-free level of SHS exposure, and that eliminating smoking in indoor spaces is the only way to fully protect nonsmokers from SHS exposure [1]. Over the past two decades, substantial progress has been achieved in implementing comprehensive smoke-free laws prohibiting smoking in all indoor workplaces and public places, including restaurants and bars, at both the local and state levels. Across the U.S., the number of local level comprehensive smoke-free laws increased from two in 1993 to more than 600 in 2014, while the number of statewide laws increased from one in 2002 to 26 and the District of Columbia in 2014 [3, 4]. However, while adoption of local level comprehensive smoke-free laws continues, the adoption of statewide laws has stalled in recent years and residents of 24 states remain unprotected by such laws and, thus, are susceptible to SHS exposure in many indoor public spaces [5]. Approximately 58 million Americans, including 15 million children, continue to be exposed to SHS [6].

Research on public perception toward the harmful effects of SHS is growing [7, 8] and studies have revealed that sociodemographic correlates may contribute to harm perception [2] and the use of noncombustible tobacco products [9–11]. Levels of support for smoke-free public places have been shown to vary by cigarette smoking status, experience with smoke-free environments, knowledge about the harmfulness of SHS exposure, and other factors [12–18]. Changes in attitudes about the acceptability of SHS exposure and the benefits of smoke-free environments both contribute to, and are further accelerated by, the publicity, education, debate, and experiences that generally accompany the adoption of smoke-free laws [1, 19]. State and local smoke-free laws typically receive high levels of public support, with this support increasing over time as people become accustomed to the protections afforded by these laws [20].

Awareness of the adverse health effects of SHS exposure is an especially important factor shaping public attitudes towards smoke-free policies [21]. Research indicates that increased knowledge about the harmfulness of SHS is associated with greater efforts to minimize exposure [22, 23], reduced SHS exposure among both smokers and nonsmokers [22–24], and adoption of smoke-free home rules [25, 26]. Increased awareness of the adverse health effects of SHS exposure is also associated with lower smoking initiation among youth [27] and more favorable attitudes toward smoke-free environments [1, 19, 28].

Past research suggests that many U.S. adults recognize the dangers of SHS exposure. For example, using data from 2001, McMillen and colleagues reported that 95.1 % of U.S. adults believed that inhaling smoke from a parent’s cigarette caused any level of harm to infants and children, with lower prevalence among smokers (89.5 %) than nonsmokers (96.7 %) [13]. These findings were consistent with studies showing smokers are less likely to perceive that SHS exposure is harmful [3, 24]. Similarly, a Gallup poll conducted during July 2014 found that 57 % of U.S. adults viewed SHS as ‘very harmful’ [29]. However, to date, no published studies have assessed potential variations in perceived harm from SHS exposure comprehensively across states, tobacco use status, smoke-free law coverage, and certain sociodemographic factors. Thus, we assessed the prevalence and sociodemographic correlates of perceptions that exposure to SHS is ‘very harmful’ both overall and by state and tobacco use status among a nationally representative sample of U.S. adults.

## Methods

### Sample

Data came from the 2009–2010 National Adult Tobacco Survey (NATS), a national landline and cellular telephone survey of noninstitutionalized civilian adults aged 18 years or older residing in the 50 U.S. states and District of Columbia [30]. The study design has been described in detail elsewhere, including cognitive testing of the employed measures [31]. In brief, the 2009–2010 NATS used a stratified, multistage probability design to yield data representative at both the national and state levels. For the landline component, each state was allocated an equal target sample size (*n* = 1863) to ensure adequate precision for state estimates. For the cellular telephone component, each state was allocated a sample size in proportion to its population (range: 255–24,100). Louisiana, New Jersey, and Oklahoma added to their landline and cellular telephone target sample size; Delaware, Georgia, Iowa, North Dakota, Pennsylvania, South Carolina, and Virginia added to their landline target sample size.

The sample design consists of a dual frame Random Digit Dialed sample, with independent samples drawn from landline and cell phone fames [31]. Respondent selection varied by telephone type. For the landline numbers, one adult was randomly selected from each eligible household. For cellular telephone numbers, the adult reached was selected if a cellular telephone was the only way they could be reached at home. Interviews were administered from October 20, 2009 to February 28, 2010, and were conducted in both English and Spanish. In total, 118,581 interviews were conducted (landline = 110,634 and cellular = 7947). The Council of American Survey and Research Organizations (CASRO) overall response rate, defined as the number of completed interviews divided by the number of eligible respondents in the sample, was 37.6 % (landline = 40.4 % and cellular = 24.9 %) [32]. The overall cooperation rate, defined as the number of completed interviews divided by the number of eligible respondents who were successfully reached by an interviewer, was 62.3 % (landline = 61.9 % and cellular = 68.7 %) [32]. State-specific CASRO response rates ranged from 28.2 % in New Jersey to 49.3 % in Vermont, while cooperation rates ranged from 52.9 % in Louisiana to 72.4 % in Vermont.

### Measures

#### Harm perceptions

Perceptions about the harm of SHS exposure were assessed by the question, “Do you think that breathing smoke from other people’s cigarettes or from other tobacco products is…?”, with the following response options: ‘not at all harmful to one’s health’, ‘somewhat harmful to one’s health’, and ‘very harmful to one’s health’. In this analysis, respondents who answered ‘very harmful to one’s health’ were considered to perceive SHS as ‘very harmful’.

#### Current tobacco product use

Tobacco product use was determined by respondents’ answers to questions on use of six products (cigarettes, cigars/cigarillos/little cigars, water pipes, pipes, chew/snuff/dip, and snus); respondents were classified into four categories: 1) combustible only use; 2) noncombustible only use; 3) combustible and noncombustible use; 4) and no tobacco use. ‘Combustible only use’ was defined as a respondent who reported smoking at least 100 cigarettes during their lifetime, and now smoked ‘every day’ or ‘some days’ and/or used cigars/cigarillos/little cigars, water pipes, or pipes on ≥1 day during the past 30 days, but did not use a noncombustible product. ‘Noncombustible only use’ was defined as a respondent who reported using chewing tobacco/snuff/dip or snus on ≥1 day during the past 30 days, but did not use a combustible product. ‘Combustible and noncombustible use’ was defined as a respondent who reported smoking at least 100 cigarettes during their lifetime and now smoked ‘every day’ or ‘some days’ and/or used cigars/cigarillos/little cigars, water pipes, pipes, chewing tobacco/snuff/dip, or snus on ≥1 day during the past 30 days. ‘No tobacco use’ was defined as a respondent who did not currently use combustible or noncombustible tobacco.

#### Comprehensive smoke-free law coverage

Comprehensive smoke-free law coverage was determined using the American Nonsmokers’ Rights Foundation (ANRF) U.S. Tobacco Control Laws Database^©^, which tracks U.S. municipal, county, and state laws relating to tobacco [33]. Laws included in this database are identified through systematic scanning of tobacco control publications, Web sites and e-mail discussion lists, biannual solicitation of information from tobacco control professionals, and partnerships. Identified tobacco control laws are coded by senior staff members at ANRF; the online legal research database of state and/or local comprehensive smoke-free laws are regularly updated quarterly. For the purposes of this study, respondents were categorized according to the proportion of individuals in their state covered by a state and/or local comprehensive smoke-free law as of July 1, 2010 [33]. A comprehensive smoke-free law was defined as an ordinance or regulation that prohibits smoking in all indoor areas of non-hospitality workplaces, restaurants, and freestanding bars, including attached bars or separately ventilated rooms with no size exemptions. For the purposes of analysis, comprehensive smoke-free laws were categorized as follows: 100 % (statewide law); 20–99 %; 1–19 %; 0 % (no local or statewide laws). These categories were selected based on the population distribution of coverage across states so as to ensure that statistically reliable comparisons could be made across categories.

#### Sociodemographic characteristics

Sociodemographic characteristics of respondents that were assessed included: sex (male or female); age group (18–24, 25–44, 45–64, or ≥65 years); race/ethnicity (non-Hispanic white, non-Hispanic black, non-Hispanic Asian, non-Hispanic American Indian/Alaska native, non-Hispanic Native Hawaiian/Pacific Islander, non-Hispanic multirace, non-Hispanic other, or Hispanic); educational attainment (0–12 years [no diploma], Graduate Equivalency Degree (GED), high school graduate, some college [no degree], associate degree, undergraduate degree, or graduate degree); marital status (single/separated/divorced/widowed or married/living with a partner); annual household income (<$20,000, $20,000–$49,999, $50,000–$99,999, ≥$100,000, or unspecified); U.S. Census region (Northeast, Midwest, South, or West); sexual orientation (heterosexual/straight, lesbian/gay/bisexual/transgender [LGBT], or unspecified); and whether the respondent lived in a household with children ≤17 years old (yes or no).

### Analyses

Data were analyzed using SAS-Callable SUDAAN 10 (SAS Institute Inc., Research Triangle Park, NC) and weighted to adjust for the differential probabilities of selection and response [31]. The landline data were first weighted by the inverse of the probability of selection of the telephone number, a nonresponse adjustment, and adjustments for the number of landlines and the number of eligible subjects in the household. The cellular telephone data were first weighted by the inverse of the probability of selection of the telephone number and a nonresponse adjustment. Next, the data were poststratified by state according to the distribution of demographic variables (sex, age, race/ethnicity, marital status, and educational attainment) and telephone type. For states with a small number of cellular telephone respondents, the use of both landline and cellular telephone data resulted in a large unequal weighting effect and, therefore, large estimated variances of survey estimates and small effective sample sizes. As a result, the national and state estimates were calculated differently. For national estimates, we included both cellular telephone and landline respondents. For state estimates, cellular telephone respondents were included only for the 12 states that had a cellular telephone sample of 200 or more respondents (California, Florida, Georgia, Illinois, Louisiana, New Jersey, New York, North Carolina, Ohio, Oklahoma, Pennsylvania, and Texas).

Descriptive analyses, including point estimates and 95 % confidence intervals (CI), were calculated for each respondent characteristic—overall, by state, and by tobacco use status. Differences between estimates were considered statistically significant if 95 % CIs did not overlap. Additionally, a binary logistic regression model was constructed with perceptions about SHS exposure being ‘very harmful’ as the dependent variable; independent variables included: sex, age group, race/ethnicity, educational attainment, marital status, annual household income, U.S. region, sexual orientation, whether respondents had children ≤17 years old living in the household, comprehensive smoke-free law coverage, and current tobacco use. Separate logistic regression models were constructed to predict perceived harm from SHS exposure among each respective category of tobacco users (i.e., combustible only use, noncombustible only use, combustible and noncombustible use, no tobacco use), each of which adjusted for the same covariates as the overall model (excluding tobacco use).

## Results

### Perception of harm from secondhand smoke exposure, overall

Nationally, 64.5 % of adults reported that SHS exposure was ‘very harmful’, 31.5 % reported it was ‘somewhat harmful,’ and 4.0 % of reported it was ‘not at all harmful’ (Table 1). The unadjusted prevalence of perceiving that SHS exposure was ‘very harmful’ was higher among females (72.5 %) compared to males (56.3 %); among respondents with an unspecified sexual orientation (79.2 %) compared to heterosexual/straight individuals (64.5 %) and LGBT individuals (59.5 %); and among respondents with children living in the household (68.2 %) compared to those without children living in the household (62.0 %) (*p* < 0.05). The prevalence of perceiving that SHS exposure was ‘very harmful’ was lower among residents of states in the Midwest (60.5 %) compared to those in other regions (South = 64.5 %; Northeast = 66.0 %; West = 67.5 %) (*p* < 0.05).

**Table 1:** Percentage of U.S. adults who think breathing smoke from other people’s cigarettes or tobacco products causes harm, by selected characteristics—National Adult Tobacco Survey (NATS) 2009–2010

	Unweighted frequency	Not at all harmful to one’s health	Somewhat harmful	Very harmful
Characteristics	*n*	% (95 % CI)	% (95 % CI)	% (95 % CI)
Overall	97,805	4.0 (3.7–4.3)	31.5 (30.9–32.1)	64.5 (63.9–65.2)
Sex				
Male	38,659	**5.3 (4.8**–**5.7)**	**38.4 (37.4**–**39.4)**	56.3 (55.3–57.4)
Female	59,146	2.8 (2.5–3.1)	24.8 (24.1–25.5)	**72.5 (71.7**–**73.2)**
Age (years)				
18–24	4780	2.6 (2.0–3.3)	32.4 (30.4–34.5)	64.9 (62.9–67.0)
25–44	25,915	3.0 (2.6–3.5)	30.9 (29.8–32.0)	66.0 (64.9–67.2)
45–64	41,968	4.9 (4.4–5.3)	33.6 (32.7–34.5)	61.5 (60.5–62.4)
≥65	25,142	5.8 (5.2–6.4)	27.2 (26.1–28.3)	67.0 (65.8–68.2)
Race/ethnicity				
White, non-Hispanic	81,517	4.2 (3.9–4.4)	35.7 (35.1–36.3)	60.1 (59.5–60.8)
Black, non-Hispanic	7244	3.0 (2.3–3.6)	21.5 (19.7–23.3)	75.5 (73.7–77.4)
Asian, non-Hispanic	1645	^a^	21.0 (15.6–26.5)	75.9 (70.3–81.5)
AI/AN, non-Hispanic	1495	7.5 (4.9–10.0)	28.4 (23.5–33.3)	64.1 (58.9–69.3)
NH/PI, non-Hispanic	399	^a^	28.0 (18.5–37.4)	66.5 (56.6–76.4)
Non-Hispanic, multirace	1220	3.7 (2.1–5.4)	28.4 (23.5–33.2)	67.9 (62.8–73.0)
Non-Hispanic, other	481	^a^	23.0 (15.4–30.5)	69.8 (61.2–78.3)
Hispanic	3804	3.8 (2.7–4.8)	20.9 (18.5– 23.3)	75.3 (72.8–77.9)
Education				
0–12 years (no diploma)	6694	5.5 (4.5–6.4)	27.2 (24.9–29.5)	67.4 (65.0–69.8)
GED	1696	6.1 (3.9–8.4)	33.8 (29.7–37.8)	60.1 (55.9–64.4)
High school graduate	21,078	4.9 (4.3–5.5)	32.5 (31.3–33.7)	62.6 (61.3–63.9)
Some college (no degree)	15,778	3.9 (3.4–4.5)	32.5 (31.1–33.9)	63.6 (62.2–65.0)
Associate degree	14,241	3.4 (2.8–3.9)	31.1 (29.7–32.5)	65.5 (64.1–67.0)
Undergraduate degree	23,148	2.3 (2.0–2.6)	33.2 (32.0–34.3)	64.6 (63.4–65.7)
Graduate degree	15,170	2.2 (1.8–2.7)	30.8 (29.5–32.2)	66.9 (65.6–68.3)
Marital status				
Married/partnered	59,208	3.7 (3.4–4.0)	30.9 (30.1–31.6)	65.4 (64.6–66.2)
Single/separated/divorced or widowed	38,597	4.4 (4.0–4.8)	32.3 (31.3–33.3)	63.3 (62.2–64.3)
Annual household income				
<$20,000	11,039	**6.1 (5.2**–**7.0)**	25.9 (24.1–27.6)	68.1 (66.2–70.0)
$20,000–$49,999	31,555	4.0 (3.6–4.4)	31.4 (30.3–32.5)	64.6 (63.4–65.7)
$50,000–$99,999	33,335	3.8 (3.4–4.3)	33.0 (32.0–34.1)	63.1 (62.1–64.2)
≥$100,000	18,357	3.0 (2.4–3.6)	34.2 (32.9–35.5)	62.8 (61.4–64.2)
Unspecified	3519	3.1 (2.1–4.0)	27.8 (24.7–30.9)	69.1 (65.9–72.3)
U.S. region				
Northeast	17,685	3.3 (2.8–3.8)	30.7 (29.5–32.0)	66.0 (64.7–67.3)
Midwest	20,255	4.4 (3.9–4.9)	**35.1 (34.0**–**36.2)**	**60.5 (59.4**–**61.7)**
South	38,595	4.3 (3.9–4.8)	31.3 (30.3–32.3)	64.4 (63.3–65.4)
West	21,270	3.7 (3.1–4.3)	28.8 (27.2–30.3)	67.5 (65.9–69.1)
Sexual orientation				
Heterosexual/straight	94,698	4.0 (3.7–4.2)	31.5 (30.9–32.2)	64.5 (63.8–65.1)
LGBT	2180	3.6 (2.3–4.8)	**36.9 (32.6**–**41.2)**	59.5 (55.2–63.9)
Unspecified	927	6.9 (3.4–10.3)	13.9 (9.6–18.3)	**79.2 (73.8**–**84.6)**
Children ≤17 years old living in household				
Yes	30,626	3.0 (2.6–3.5)	28.8 (27.7–29.8)	**68.2 (67.1**–**69.3)**
No	67,179	**4.7 (4.4**–**5.0)**	**33.4 (32.6**–**34.1)**	62.0 (61.2–62.7)
Tobacco use^c^				
Combustible only	13,665	8.7 (7.8–9.6)	43.5 (41.8–45.1)	47.9 (46.2–49.5)
Noncombustible only	527	5.2 (2.4–8.0)	32.6 (25.3–40.0)	62.1 (54.6–69.7)
Combustible & Noncombustible	1080	9.2 (6.5–12.0)	50.1 (44.8–55.4)	40.7 (35.5–46.0)
No tobacco use	49,778	2.0 (1.7–2.2)	21.5 (20.7–22.4)	76.5 (75.6–77.3)
Population coverage of smoke-free laws^b^				
100 % state laws	46,285	4.0 (3.6–4.4)	31.1 (30.3–31.9)	64.9 (64.1–65.8)
20–99 % state laws	15,902	4.3 (3.6–4.9)	33.6 (31.8–35.4)	62.1 (60.3–64.0)
1–19 % state laws	16,333	3.7 (3.1–4.3)	29.1 (27.5–30.7)	67.2 (65.5–68.9)
0 % state or local laws	17,785	4.2 (3.7–4.7)	33.8 (32.6–35.0)	62.0 (60.8–63.2)

Note: Bold values indicate statistical significance (*p* < 0.05) when compared to all other categories for the respective characteristic

*Abbreviations: AI/AN* American Indian/Alaska Native, *NH/PI* Native Hawaiian/Pacific Islander, *GED* Graduate Equivalency Degree, *LGBT* Lesbian, Gay, Bisexual, or Transgender

^a^Estimate excluded due to relative standard error >30 %

^b^Population coverage of smoke-free laws was based on the American Nonsmokers’ Rights Foundation U.S. Tobacco Control Laws Database^©^ and represent the percent of the population covered by state and/or local comprehensive smoke-free laws as of July 1, 2010

^c^Combustible only use is defined as those who currently smoke cigarettes, pipes, hookah, or cigars and do not use snus or chew/snuff/dip. Noncombustible only use is defined as those who use snus or chew/snuff/dip and do not currently smoke cigarettes, pipes, hookah or cigars. Combustible and noncombustible users are those who use both combustible and noncombustible tobacco products

### Perception of harm from secondhand smoke exposure, by state

Table 2 shows the prevalence of harm perceptions toward SHS among adults by state. The prevalence of those who perceived SHS exposure to be ‘very harmful’ to health ranged from 73.5 % in Utah to 53.7 % in Kentucky; the prevalence of those who considered exposure to be ‘somewhat harmful’ ranged from 39.5 % in Kentucky to 25.3 % in Florida; and the prevalence of those who considered exposure to be ‘not at all harmful’ ranged from 6.8 % in Kentucky to 2.4 % in Delaware.

**Table 2:** Percentage of U.S. adults who think breathing smoke from other people’s cigarettes or other tobacco products causes harm, by state—National Adult Tobacco Survey (NATS) 2009–2010

State	Unweighted frequency	Not at all harmful to one’s health	Somewhat harmful	Very harmful
	*n*	% (95 % CI)	% (95 % CI)	% (95 % CI)
100 % of population covered by a comprehensive smoke-free law^a^				
Arizona	1539	5.7 (3.7–7.7)	28.2 (24.2–32.1)	66.1 (61.9–70.4)
Colorado	1634	3.4 (2.2–4.5)	35.7 (32.0–39.5)	60.9 (57.1–64.7)
Delaware	1585	2.4 (1.3–3.4)	32.0 (25.2–38.7)	65.6 (59.0–72.3)
District of Columbia	1427	^b^	30.0 (22.1–38.0)	67.1 (59.1–75.1)
Florida^c^	1859	5.6 (3.8–7.5)	25.3 (22.7–27.9)	69.1 (66.2–71.9)
Hawaii	1509	4.2 (2.0–6.4)	29.5 (24.3–34.8)	66.3 (60.8–71.7)
Illinois^c^	1698	4.0 (2.8–5.2)	33.3 (30.2–36.4)	62.7 (59.6–65.9)
Iowa	1768	2.7 (1.8–3.7)	36.0 (31.8–40.3)	61.2 (57.0–65.5)
Louisiana^c^	5164	4.7 (3.8–5.6)	27.3 (25.3–29.4)	67.9 (65.8–70.0)
Maine	1723	3.7 (2.3–5.2)	31.4 (26.6–36.2)	64.9 (60.0–69.7)
Maryland	1548	2.4 (1.3–3.5)	34.3 (30.7–37.9)	63.3 (59.7–67.0)
Massachusetts^c^	1563	3.7 (2.3–5.2)	33.1 (29.7–36.4)	63.2 (59.8–66.6)
Minnesota	1563	5.2 (2.9–7.4)	38.4 (34.8–41.9)	56.4 (52.8–60.1)
Montana	1579	5.2 (2.7–7.7)	31.7 (27.3–36.1)	63.1 (58.5–67.8)
Nebraska	1587	5.2 (2.3–8.0)	37.4 (31.4–43.4)	57.4 (51.4–63.4)
Nevada	1554	5.4 (3.3–7.5)	34.8 (29.7–40.0)	59.8 (54.6–65.0)
New Jersey^c^	3308	3.4 (2.4–4.4)	32.1 (29.8–34.4)	64.5 (62.1–66.8)
New Mexico	1488	3.9 (1.8–6.0)	27.6 (22.8–32.4)	68.5 (63.6–73.5)
New York^c^	1839	2.5 (1.6–3.4)	27.1 (24.4–29.8)	70.4 (67.6–73.2)
Ohio^c^	1811	4.7 (3.4–6.0)	35.3 (32.6–38.1)	59.9 (57.1–62.7)
Oregon	1697	3.4 (1.8–5.0)	33.9 (30.5–37.3)	62.7 (59.1–66.2)
Rhode Island	1566	^b^	30.1 (26.0–34.2)	65.4 (61.0–69.8)
Utah	1796	2.6 (1.5–3.7)	23.9 (20.5–27.4)	73.5 (69.9–77.0)
Vermont	1734	2.0 (1.1–3.0)	32.0 (26.3–37.8)	65.9 (60.1–71.7)
20–99 % of population covered by comprehensive smoke-free law				
Washington	1746	3.3 (1.9–4.8)	33.3 (30.4–36.3)	63.3 (60.2–66.4)
Alaska	1566	2.6 (1.4–3.8)	28.5 (21.6–35.5)	68.9 (61.8–75.9)
Indiana	1701	4.8 (3.3–6.4)	35.0 (31.8–38.2)	60.2 (56.9–63.4)
Kansas	1602	4.6 (2.3–7.0)	35.2 (30.6–39.8)	60.2 (55.4–64.9)
Kentucky	1532	6.8 (4.3–9.2)	39.5 (34.6–44.5)	53.7 (48.7–58.7)
North Dakota	1850	^b^	30.5 (21.1–39.9)	65.6 (56.2–75.1)
South Carolina	4165	4.2 (2.6–5.9)	31.8 (28.1–35.5)	63.9 (60.1–67.7)
Texas^c^	1942	3.6 (2.7–4.6)	32.7 (29.6–35.7)	63.7 (60.6–66.8)
West Virginia	1544	4.6 (2.7–6.5)	30.3 (26.2–34.3)	65.1 (60.9–69.4)
1–19 % of population covered by a comprehensive smoke-free laws				
Alabama	1611	3.7 (2.3–5.2)	29.4 (25.5–33.3)	66.8 (62.7–70.9)
Arkansas	2384	^b^	35.0 (27.6–42.4)	56.9 (49.5–64.4)
California^c^	2159	3.4 (2.3–4.4)	26.1 (23.4–28.8)	70.5 (67.7–73.4)
Georgia^c^	3976	3.4 (2.5–4.2)	29.0 (26.6–31.5)	67.6 (65.0–70.1)
Mississippi	1466	3.0 (1.9–4.1)	27.0 (21.3–32.6)	70.1 (64.3–75.8)
Missouri	1666	3.8 (2.6–5.0)	38.2 (34.9–41.4)	58.0 (54.7–61.4)
Wisconsin	1619	4.3 (2.5–6.0)	35.4 (31.8–39.0)	60.4 (56.7–64.1)
Wyoming	1452	^b^	37.4 (30.0–44.8)	58.2 (50.9–65.5)
0 % of population covered by comprehensive smoke-free law				
Connecticut	1500	3.1 (1.9–4.3)	30.3 (26.6–34.0)	66.6 (62.8–70.3)
Idaho	1551	3.3 (1.5–5.0)	31.5 (25.7–37.3)	65.2 (59.3–71.1)
Michigan	1656	4.6 (3.1–6.2)	33.4 (30.1–36.8)	61.9 (58.5–65.4)
New Hampshire	1638	^b^	32.0 (27.8–36.3)	64.2 (59.7–68.7)
North Carolina^c^	1665	4.8 (3.3–6.2)	36.5 (33.2–39.8)	58.7 (55.4–62.1)
Oklahoma^c^	3098	3.8 (3.0–4.7)	30.7 (28.7–32.8)	65.4 (63.3–67.5)
Pennsylvania^c^	2814	4.1 (3.1–5.1)	33.9 (31.6–36.2)	62.0 (59.7–64.3)
South Dakota	1734	3.1 (2.0–4.2)	30.4 (26.4–34.5)	66.4 (62.3–70.5)
Tennessee	1617	4.6 (3.2–6.0)	33.4 (29.7–37.0)	62.0 (58.3–65.8)
Virginia	2012	3.6 (2.5–4.7)	33.7 (30.8–36.6)	62.7 (59.8–65.7)
National	97,805	4.0 (3.7–4.3)	31.5 (30.9–32.1)	64.5 (63.9–65.2)

^a^Population coverage of smoke-free laws was based on the American Nonsmokers’ Rights Foundation U.S. Tobacco Control Laws Database^©^ and represent the percent of the population covered by state and/or local comprehensive smoke-free laws as of July 1, 2010

^b^Relative SE >30 %

^c^Estimates calculated among both landline and cellular telephone respondents. All other state estimates were calculated among landline respondents only

### Adjusted odds of perceived harm, overall

Overall, the adjusted odds of perceiving SHS to be ‘very harmful’ were higher among females (adjusted odds ratio [AOR] = 1.6, 95 % CI = 1.4–1.7) compared to men; among non-Hispanic Blacks (AOR = 2.0, 95 % CI = 1.7–2.3), non-Hispanic Asians (AOR = 1.7, 95 % CI = 1.1–2.5), non-Hispanic American Indian/Alaska Natives (AOR = 1.5, 95 % CI = 1.1–2.0), non-Hispanic others (AOR = 2.1, 95 % CI = 1.2–3.8), and Hispanics (AOR = 1.8, 95 % CI = 1.5–2.2) compared to non-Hispanic whites; among respondents with children living in the household (AOR = 1.3, 95 % CI = 1.1–1.4) compared to respondents without children living in the household; and respondents covered by a 100 % smoke-free law (AOR = 1.1, 95 % CI = 1.1–1.3) compared to those living in states with less coverage (Table 3).

**Table 3:** Percent and adjusted odds ratios of U.S. adults who think breathing smoke from other people’s cigarettes or other tobacco products was ‘very harmful’ overall by tobacco use status^a^ and selected characteristics—National Adult Tobacco Survey (NATS) 2009–2010

	Combustible only		Noncombustible only		Combustible & Noncombustible		No tobacco use		Overall	
	(*n* = 5648)		(*n* = 297)		(*n* = 441)		(*n* = 38,074)		(*n* = 44,460)	
Characteristics	%	AOR (95 % CI)^b^	%	AOR (95 % CI)^b^	%	AOR (95 % CI)^b^	%	AOR (95 % CI)^b^	%	AOR (95 % CI)^b^
Overall	47.9 (46.2–49.5)	**0.3 (0.2**–**0.4)**	62.1 (54.3–69.4)	**0.7 (0.5**–**0.9)**	40.7 (35.6–46.0)	**0.3 (0.2**–**0.4)**	76.5 (75.6–77.3)	1.00	67.3 (66.5–68.0)	^e^
Sex										
Male	45.1 (42.6–47.7)	1.00	59.6 (51.0–67.6)	1.00	40.6 (35.1–46.2)	1.00	70.3 (68.4–72.2)	1.00	58.7 (57.2–60.2)	1.00
Female	50.8 (48.7–52.9)	**1.3 (1.1**–**0.5)**	77.3 (61.5–87.9)	1.6 (0.6–4.0)	42.1 (27.4–58.4)	1.0 (0.5–2.1)	79.2 (78.4–80.1)	**1.7 (1.5**–**1.9)**	72.8 (72.0–73.6)	**1.6 (1.4**–**1.7)**
Age (years)										
18–24	55.2 (50.5–59.8)	1.00	70.5 (49.5–85.4)	1.00	44.3 (34.6–54.4)	1.00	74.5 (71.6–77.1)	1.00	66.3 (63.9–68.7)	1.00
25–44	53.8 (51.0–56.5)	0.9 (0.7–1.2)	59.3 (47.2–70.4)	0.4 (0.2–1.2)	37.9 (31.0–45.2)	0.6 (0.4–1.0)	78.0 (76.4–79.6)	1.0 (0.8–1.3)	68.8 (67.4–70.2)	1.0 (0.8–1.1)
45–64	39.8 (37.5–42.1)	**0.6 (0.5**–**0.8)**	59.9 (48.1–70.6)	0.6 (0.2–1.6)	39.0 (28.7–50.4)	0.7 (0.4–1.3)	75.8 (74.6–77.0)	1.0 (0.9–1.2)	64.6 (63.4–65.7)	**0.8 (0.7**–**0.9)**
≥65	33.8 (29.7–38.3)	**0.5 (0.4**–**0.6)**	65.1 (45.4–80.8)	0.4 (0.1–1.6)	55.9 (34.9–74.9)	1.7 (0.7–4.3)	76.0 (74.6–77.4)	1.1 (0.0–1.4)	70.0 (68.5–71.4)	0.9 (0.8–1.1)
Race/ethnicity										
White, non-Hispanic	41.8 (40.0–43.5)	1.00	59.6 (51.2–67.6)	1.00	35.2 (30.1–40.8)	1.00	73.0 (72.1–73.8)	1.00	62.6 (61.8–63.4)	1.00
Black, non-Hispanic	60.4 (56.0–64.7)	**2.1 (1.7**–**2.6)**	64.1 (37.1–84.4)	1.1 (0.4–3.2)	69.4 (45.4–86.1)	6.3 (2.2–8.1)	83.1 (80.8–85.3)	**1.9 (1.6**–**2.3)**	76.3 (74.2–78.3)	**2.0 (1.7**–**2.3)**
Asian, non-Hispanic	48.0 (33.4–63.0)	1.2 (0.6–2.3)	^e^	^e^	12.6 (1.3–61.4)	0.2 (0.0–3.4)	82.7 (73.8–89.1)	**1.8 (1.1**–**3.0)**	78.3 (70.7–84.3)	**1.7 (1.1**–**2.5)**
AI/AN, non-Hispanic	53.9 (43.4–64.0)	**1.6 (1.1**–**2.4)**	^d^	0.2 (0.0–2.1)	^d^	1.33 (0.6–3.1)	81.1 (71.0–88.3)	1.6 (0.9–2.7)	65.0 (58.4–71.0)	**1.5 (1.1**– **2.0)**
NH/PI, non-Hispanic	58.2 (39.6–74.8)	1.7 (0.7–3.9)	^e^	^e^	65.1 (17.3–94.3)	2.9 (0.4–21.0)	76.2 (59.7–87.4)	1.1 (0.5–2.5)	70.4 (58.5–80.0)	1.4 (0.8–2.5)
Non-Hispanic, multirace	47.5 (36.4–58.8)	1.1 (0.7–1.7)	^d^	0.6 (0.1–3.5)	^d^	2.9 (0.7–11.9)	80.4 (73.2–86.0)	1.5 (1.0–2.3)	66.5 (60.2–72.3)	1.3 (1.0–1.7)
Non-Hispanic, other	57.9 (36.7–76.6)	1.7 (0.7–4.1)	96.5 (75.5–99.6)	50.9 (5.0–514.6)	78.5 (35.3–96.1)	8.2 (0.3–202.5)	86.7 (72.8–94.1)	2.3 (1.0–5.6)	74.8 (63.6–83.5)	**2.1 (1.2**–**3.8)**
Hispanic	63.8 (56.8–70.3)	**1.9 (1.4**–**2.7)**	86.3 (45.4–98.0)	7.8 (0.6–109.8)	96.7 (85.7–99.3)	3.3 (1.4–7.8)	82.7 (79.5–85.5)	**1.8 (1.4**–**2.2)**	77.3 (74.3–80.1)	**1.8 (1.5**–**2.2)**
Education										
0–12 years (no diploma)	56.5 (51.9–61.0)	1.00	73.5 (59.0–84.2)	1.00	45.3 (32.7–58.5)	1.00	77.9 (74.5–81.0)	1.00	68.8 (66.1–71.4)	1.00
GED	50.4 (42.7–58.0)	0.8 (0.6–1.2)	^d^	0.6 (0.1–3.8)	49.3 (28.8–70.0)	1.8 (0.7–4.6)	73.7 (65.9–80.2)	0.8 (0.6–1.2)	59.2 (53.8–64.4)	0.9 (0.7–1.2)
High school graduate	44.7 (41.8–47.6)	**0.7 (0.6**–**0.9)**	68.3 (54.4–79.6)	0.7 (0.3–2.0)	36.9 (29.1–45.3)	0.9 (0.5–1.7)	75.3 (73.5–77.0)	1.0 (0.8–1.3)	64.7 (63.2–66.2)	0.9 (0.8–1.0)
Some college (no degree)	45.3 (41.8–48.9)	**0.7 (0.6**–**0.9)**	48.3 (28.3–68.8)	0.4 (0.1–1.2)	41.9 (30.8–53.8)	1.1 (0.5–2.3)	75.7 (73.8–77.5)	1.0 (0.8–1.3)	66.0 (64.3–67.7)	0.9 (0.74–1.0)
Associate degree	49.3 (45.4–53.3)	0.9 (0.7–1.1)	39.7 (22.3–60.3)	**0.1 (0.0**–**0.5)**	34.4 (23.9–46.8)	1.0 (0.5–2.2)	79.1 (77.4–80.7)	1.3 (1.0–1.6)	69.5 (67.8–71.2)	1.0 (0.9–1.2)
Undergraduate degree	41.0 (37.2–44.8)	**0.7 (0.5**–**0.9)**	58.6 (36.8–77.5)	0.5 (0.1–1.8)	40.7 (26.3–57.0)	1.4 (0.6–3.6)	75.7 (74.2–77.1)	1.1 (0.9–1.4)	68.9 (67.5–70.3)	0.9 (0.8–1.1)
Graduate degree	40.4 (34.7–46.4)	**0.7 (0.5**–**0.9)**	^d^	**0.1 (0.0**–**0.7)**	^d^	1.0 (0.3–3.3)	76.6 (75.0–78.2)	1.1 (0.9–1.4)	71.6 (69.9–73.2)	1.0 (0.8–1.1)
Marital status										
Married/partnered	48.4 (46.1–50.7)	1.1 (1.0–1.3)	95.4 (89.7–98.0)	2.0 (0.9–4.1)	41.2 (34.0–48.9)	1.0 (0.6–1.5)	76.7 (75.7–77.8)	1.1 (1.0–1.2)	68.6 (67.6–69.6)	1.1 (1.1–1.2)
Single/separated/ divorced or widowed	47.4 (45.0–49.7)	1.00	94.0 (88.5–97.0)	1.00	40.3 (33.2–47.7)	1.00	76.1 (74.7–77.5)	1.00	65.6 (64.4–66.8)	1.00
Annual household income										
<$20,000	53.7 (49.8–57.4)	1.00	63.7 (54.5–71.9)	1.00	40.3 (27.2–55.0)	1.00	81.2 (78.8–83.4)	1.00	69.2 (67.0–71.3)	1.00
$20,000–$49,999	47.9 (45.3–50.6)	0.8 (0.7–1.0)	60.1 (46.7–72.2)	0.5 (0.2–1.5)	37.4 (29.6–45.8)	1.0 (0.5–1.9)	76.4 (74.7–78.0)	**0.8 (0.7**–**0.9)**	66.2 (64.8–67.6)	**0.8 (0.7**–**0.9)**
$50,000–$99,999	42.3 (39.2–45.5)	**0.7 (0.6**–**0.9)**	62.1 (54.3–69.4)	**0.3 (0.1**–**0.9)**	47.7 (38.0–57.5)	1.8 (0.9–3.8)	75.7 (74.4–77.0)	**0.8 (0.7**–**0.9)**	66.8 65.5–68.1)	**0.8 (0.7**–**0.9)**
≥$100,000	44.5 (39.3–49.8)	0.8 (0.6–1.1)	77.5 (59.8–88.8)	0.3 (0.1–1.2)	30.9 (19.6–45.0)	0.7 (0.3–1.6)	74.8 (73.0–76.5)	**0.8 (0.6**–**0.9)**	67.4 (65.5–69.2)	**0.9 (0.7**–**0.9)**
Unspecified	58.2 (50.1–66.0)	1.1 (0.8–1.6)	^d^	**0.2 (0.0**–**0.8)**	60.6 (39.2–78.6)	2.6 (0.9–7.3)	75.3 (71.2–79.0)	0.8 (0.6–1.0)	70.8 (67.1–74.2)	0.9 (0.7–1.1)
U.S. region indicator										
Northeast	49.2 (45.7–52.8)	1.1 (0.9– 1.4)	68.6 (46.8–84.4)	3.2 (0.8–12.9)	45.1 (31.6–59.4)	1.0 (0.4–2.3)	76.2 (74.6–77.8)	0.9 (0.8–1.1)	68.4 (66.9–70.0)	1.0 (0.9–1.1)
Midwest	43.6 40.4–46.8)	0.8 (0.7–1.0)	56.1 (40.6–70.6)	1.1 (0.4–3.2)	32.6 (23.3–43.5)	0.6 (0.3–1.2)	73.3 (71.8–74.8)	**0.8 (0.7**–**0.9)**	63.1 (61.6–64.5)	**0.8 (0.7**–**0.9)**
South	48.4 45.8–50.9)	1.0 (0.8–1.2)	64.8 (53.6–74.5)	2.2 (0.8–6.2)	42.0 (34.6–49.7)	0.7 (0.4–1.3)	76.9 (75.5–78.2)	1.0 (0.8–1.1)	67.2 (65.9–68.4)	1.0 (0.8–1.1)
West	50.5 46.0–54.9)	1.00	59.5 (39.5–76.7)	1.00	45.0 (32.7–58.1)	1.00	78.9 (76.7–81.0)	1.00	70.5 (68.5–72.5)	1.00
Sexual orientation										
Heterosexual/straight	47.7 46.0–49.4)	1.00	62.0 (54.1–69.4)	1.00	41.3 (36.2–46.7)	1.00	76.4 (75.5–77.2)	1.00	67.2 (66.5–68.0)	1.00
LGBT	47.3 (39.1–55.7)	0.9 (0.6–1.2)	80.4 (4.5–97.0)	1.5 (0.1–21.1)	^d^	0.2 (0.0–1.1)	74.6 67.5–80.5)	1.0 (0.7–1.4)	60.7 (55.2–66.0)	0.9 (0.7–1.1)
Unspecified	63.7 (41.4–81.3)	1.5 (0.6–3.9)	^d^	**0.0 (0.0**–**0.5)**	81.9 (41.8–96.6)	8.1 (1.2–56.3)	83.3 (76.8–88.3)	1.3 (0.8–2.0)	80.2 (73.5–85.5)	1.3 (0.9–1.9)
Children ≤17 years old living in household										
Yes	56.7 (53.9–59.4)	**1.4 (1.2**–**1.6)**	66.1 (54.9–75.8)	**2.0 (1.1**–**4.0)**	42.8 (35.0–51.0)	1.4 (0.9–2.3)	79.1 (77.7–80.4)	**1.2 (1.1**–**1.4)**	71.5 (70.2–72.8)	**1.3 (1.1**–**1.4)**
No	41.4 (39.4–43.4)	1.00	58.9 (48.1–68.9)	1.00	38.9 (32.3–45.9)	1.00	74.6 (73.5–75.6)	1.00	64.1 (63.2–65.1)	1.00
Population coverage of smoke-free laws^c^										
100 % state laws	48.7 (46.4–51.1)	1.2 (1.0–1.4)	65.3 (52.1–76.5)	**2.9 (1.1**–**7.4)**	37.3 (29.8–45.5)	**0.5 (0.3**–**0.9)**	77.0 (75.9–78.0)	**1.2 (1.1**–**1.3)**	68.1 (67.1–69.2)	**1.1 (1.1**–**1.3)**
20–99 % state laws	48.4 (44.0–52.8)	1.1 (0.9–1.4)	69.3 (56.6–79.7)	**3.2 (1.2**–**8.3)**	39.0 (27.3–52.1)	0.6 (0.3–1.2)	78.5 (76.1–80.6)	1.2 (1.0–1.3)	69.2 (67.1–71.2)	1.1 (1.0–1.2)
1–19 % state laws	47.5 (43.2–52.0)	1.1 (0.9–1.4)	51.8 (30.2–72.7)	1.3 (0.5–3.6)	40.2 (27.8–54.0)	0.7 (0.3–1.4)	75.0 (72.3–77.5)	1.0 (0.9–1.2)	65.4 (63.1–67.6)	1.0 (0.9–1.1)
0 % state or local laws	45.2 (42.0–48.5)	1.00	57.7 (43.3–70.9)	1.00	48.1 (38.4–57.9)	1.00	73.6 (72.0–75.2)	1.00	64.0 (62.5–65.5)	1.00

*Abbreviation: AOR* adjusted odds ratio, *CI* confidence interval, *AI/AN* American Indian/Alaska Native, *NH/PI* Native Hawaiian/Pacific Islander, *GED* Graduate Equivalency Degree, *LGBT* Lesbian, Gay, Bisexual, or Transgender

^a^Combustible only was defined as those who reported smoking at least 100 cigarettes during their lifetime and who currently smoke cigarettes, and/or use cigars/cigarillos/little cigars, pipes, water pipe/hookah, and do not use chewing tobacco/snuff/dip or snus. Noncombustible only was defined as those who reported using chewing tobacco/snuff/dip or snus on ≥1 day during the past 30 days and do not currently smoke cigarettes, and/or use cigars/cigarillos/little cigars, pipes, water pipe/hookah. Combustible and noncombustible users are those who use both combustible and noncombustible tobacco products. No tobacco users are those who do not currently use any combustible or noncombustible products

^b^Adjusted odds ratios were computed using a binary logistic regression model adjusted for all covariates listed in the table; additionally, tobacco use was included in the overall model. Statistically significant odds ratios are noted in bold. For tobacco use status in the overall model, the overall prevalence and adjusted OR for combustible only users was 47.9 (46.2–49.5) AOR = 0.3 (0.2–0.4); noncombustible only users was 62.1 (54.3–69.4), AOR = 0.7 (0.5–0.9); combustible and noncombustible users was 40.7 (35.6–46.0), AOR = 0.3 (0.2–0.4); and no tobacco users was 76.5 (75.6–77.3), AOR = 1.00 (referent)

^c^Population coverage of smoke-free laws was based on the ANRF’s U.S. Tobacco Control Laws Database^©^ and represents the percent of the population covered by state and/or local comprehensive smoke-free laws as of July 1, 2010

^d^Relative SE >30 %

^e^Point estimate was 100 % and adjusted odds ratio could not be calculated due to zero cell count in nontobacco users

The odds of perceiving SHS to be ‘very harmful’ were lower among respondents aged 45–64 (AOR = 0.8, 95 % CI = 0.7–0.9) compared to respondents 18–24 years of age; those with an annual household incomes >$20,000 compared to respondents with an annual income of <$20,000; and those living in the Midwest (AOR = 0.8, 95 % CI = 0.7–0.9) compared to those in the West.

### Adjusted odds of perceived harm, by tobacco use status

Perceptions that SHS was ‘very harmful’ were highest among those who did not use tobacco (76.5 %), followed by noncombustible only users (62.1 %), combustible only users (47.9 %), and combustible and noncombustible users (40.7 %) (Table 3). By tobacco use, the odds of perceiving SHS to be ‘very harmful’ were lower among noncombustible only users (AOR = 0.7, 95 % CI = 0.5–0.9), combustible only users (AOR = 0.3, 95 % CI = 0.2–0.4), and combustible and noncombustible users (AOR = 0.3, 95 % CI = 0.2–0.4) compared to those who did not use tobacco.

Among combustible only users, odds of perceiving SHS to be ‘very harmful’ were higher among females compared to males, non-Hispanic Blacks, non-Hispanic American Indian/Alaska Natives compared to non-Hispanic whites, and among respondents with children ≤17 years old living in the household compared to respondents without children living in the household; the odds were lower among respondents ≥45 years of age compared to those 18–24 years of age, high school graduates, those with some college or an undergraduate or graduate degree compared to those with 0–12 years of education, and respondents with an annual household income of $50,000–$99,000 compared to those with a household income <$20,000.

Among noncombustible only users, the odds were higher among respondents with children ≤17 years old living in the household compared to respondents without children living in the household and those living in a state with 100 % or 20-99 % population coverage of state and/or local smoke-free laws compared to those living in states with less coverage; the odds were lower among respondents with an associates and graduate degree compared to those with 0–12 years of education, respondents with an income of $50,000–$99,000 compared to those with a household income <$20,000.

Among combustible and noncombustible users, the odds were lower among respondents residing in locations with 100 % smoke-free laws compared to those without any 100 % state/local smoke-free law coverage. Among those who did not use tobacco, the odds were higher among females, non-Hispanic Blacks, non-Hispanic Asians and Hispanics, respondents with children ≤17 years old living in the household, and those covered by 100 % smoke-free laws; the odds were lower among respondents with an annual household income of ≥$20,000 and those in the Midwest.

## Discussion

The findings from this study reveal that 96 % of U.S. adults perceive SHS exposure as harmful, with nearly two-thirds (64.5 %) perceiving it to be ‘very harmful’. The prevalence of perceiving that SHS exposure was ‘very harmful’ was significantly higher among females, racial/ethnic minorities, respondents with children ≤17 years old living in the household, and those covered by 100 % smoke-free laws. Population-level perception of SHS harm data can be an important component in understanding societal contexts for developing educational campaigns [27]. Such efforts could include public health educational initiatives and media campaigns, such as CDC’s “Tips from Former Smokers,” which included ads related to the adverse health effects of SHS exposure [34].

The present study expands upon a previous study that examined perceptions of harm from SHS exposure among youth [8]. However, in this study, we assessed the proportion of adults who perceived that SHS exposure was ‘very harmful’. The rationale for assessing high levels of perceptions toward SHS was based on the findings of the 2006 Surgeon General’s report, which concluded that there is no safe level of exposure to SHS and that the only way to fully protect the public from SHS exposure is to completely eliminate smoking in indoor public places and worksites [1]. This is the first study to examine perceptions of harm by subgroupings of tobacco use status. The findings reveal that nearly two-thirds of noncombustible only tobacco users perceived SHS as ‘very harmful’ compared to approximately half of combustible only users and two-fifths of dual users of both combustible and noncombustible tobacco. The lower levels of perceptions among dual users may be attributable to multiple factors, including greater levels of tobacco dependence among these individuals [9], lower levels of understanding of the health hazards of tobacco use [11], and increased receptivity to protobacco messaging. These findings underscore the importance of continued efforts to educate all tobacco users about the benefits of smoke-free environments as part of a comprehensive approach to reduce tobacco use and SHS exposure, particularly among combustible tobacco users and dual users of both combustible and noncombustible tobacco. These findings also underscore the importance of educating smokers on the importance of quitting smoking completely as no level of exposure to SHS is risk-free.

Variations in perceptions about the harms of SHS were also observed by sociodemographic groups. More specifically, the perception that SHS was ‘very harmful’ was higher in females, non-Hispanic racial/ethnic minorities and Hispanics, and respondents with children ≤17 years’ old living in the household. These findings are generally consistent with those from other studies [8, 35–37]. Of note, estimates of harm perceptions by age differed by current smoking status. For example, among combustible only users, the odds of perceiving SHS to be ‘very harmful’ were lower for those ≥45 years of age compared to respondents 18–24 years of age. These variations in perceptions by age could be due, in part, to varying tobacco use rates among age groups [8] or factors related to the social disapproval of tobacco use [9, 38]. Consistent with past studies, respondents who reported living with children in the household had higher odds of perceiving that SHS was harmful [2], suggesting greater receptivity toward tobacco-related health messages and understanding of the dangers of SHS among individuals with children. A study by Tan and colleagues [7] suggested that raising public awareness to information about the harmfulness of SHS influenced public perceptions. More research into the influence of public education campaigns on the health effects of SHS could further increase awareness of the harms of SHS.

Variations in perceptions about the harm of SHS were also observed across states. For example, Kentucky had the lowest prevalence of adults who believed that SHS was ‘very harmful’, while Utah, California, and New York had the highest. The higher prevalence in these states may be attributable to lower smoking rates and/or longstanding comprehensive tobacco control programs in these states [3, 39]. Conversely, the relatively lower coverage in some states could be attributable to higher smoking rates or varying norms related to the social acceptability of tobacco use [40, 41]. Improving public awareness about the harms of SHS could help inform pathways to smoke-free policy implementation. The implementation of such policies could also further enhance public knowledge of the dangers of SHS and favorable attitudes towards smoke-free environments. The adoption of smoke-free policies have been shown to increase public favorability toward smoke-free environments in public and private settings [15, 42–45]. For example, Tang and colleagues found favorability toward smoke-free bar law implantation among bar patrons and bar owners and staff [46]. Similarly, Fong and colleagues documented increased support for smoke-free environments following Ireland’s implementation of comprehensive smoke-free legislation [15]. In the U.S., Cheng and colleagues documented positive associations between comprehensive smoke-free law adoption and voluntary smoke-free home rules [43]. Thus, opportunities exist for leveraging public attitudes toward SHS to promote smoke-free environments in states not protected by statewide comprehensive smoke-free policies.

Variations in harm perception were also observed by comprehensive smoke-free law coverage. A significant association was observed between perceptions about the harm of SHS exposure and population level coverage of state and/or local smoke-free laws, overall and by noncombustible only users, combustible and noncombustible users, and no tobacco users. In general, given that perceptions about the harm of SHS exposure were higher among respondents covered by a 100 % smoke-free law compared to those not covered by such a law, it is possible this result could be attributable to the changing social norms related to the social acceptability of tobacco use and the increased prevalence of smoke-free environments [47]. These findings underscore the importance of efforts to further increase public awareness of the health effects of SHS through education campaigns, which can contribute to changes in social norms regarding the acceptability of smoking around others.

The major strength of this study is that it utilized a large, nationally representative database and assessed factors associated with perceptions of harm across tobacco use categories and levels of smoke-free law coverage. However, the study is subject to at least eight limitations. First, these data are self-reported and may be subject to recall bias. Second, these data are cross-sectional and, thus, it was not possible to assess causal relationships between harm perceptions and smoke-free law adoption. Third, the overall NATS response rate was 37.6 % and state response rates ranged from 28.2 to 49.3 %; low response rates increase the potential for bias. Fourth, in order to prevent large variances in survey estimates and small sample sizes, cellular telephone respondents were excluded from estimates for states that had fewer than 200 cellular telephone respondents. However, we included cellular telephone respondents in all national estimates and in estimates for the 12 states that had a sufficient sample size. Given that cellular phone only users are more likely to be current cigarette smokers, the omission of cellular phone only users could lead to overestimation of perceived harm from SHS for the remaining 38 states [48]. Fifth, the operational definition of perceived harm used in this study did not fully align with measures used elsewhere in the literature [49]. Moreover, the employed question asked about respondents’ beliefs about breathing smoke from other people’s cigarettes or from other tobacco products, and thus, it is not possible to determine if perception from cigarette smoke may have differed from other tobacco products. Sixth, these data are from 2009 to 2010, and thus, may not reflect more recent harm perceptions toward SHS. However, although some progress has been made in reducing secondhand smoke exposure since that period at the state and local levels, it is not expected that population-level perceptions would change considerably over this five year period. Seventh, it is possible that perceptions of harm from SHS were underestimated in states with a statewide smoke-free policy that was not classified as 100 % smoke-free due to certain exemptions, but which still protect a large proportion of the state’s population from SHS exposure; for example California has an exemption for enclosed public places and places of employment, and therefore is not categorized as have a comprehensive law. However, a supplementary analysis that treated California as having 100 % coverage did not significantly impact the currently presented estimates. Finally, it was not possible to assess perceptions of harm by frequency of tobacco product use given the manner in which current use was assessed for most products in the survey; future research is critical to further explore potential associations between harm perceptions and frequency of use of all forms of tobacco used by U.S. adults.

## Conclusion

This is the first study to assess levels of perceived harm from SHS exposure by tobacco use status and comprehensive smoke-free law coverage among a nationally representative sample of U.S. adults. The findings reveal that nearly two-thirds of all U.S. adults perceived SHS exposure to be ‘very harmful’. Efforts to increase awareness about the harms of SHS exposure and the benefits of smoke-free environments may help inform efforts to protect all Americans from SHS exposure, particularly tobacco users. Variability in perceptions about the harm of SHS was observed by tobacco use status, with prevalence being highest among adults who do not use tobacco and lowest among those who use both combustible and noncombustible tobacco. Additionally, perceptions about the harm of SHS exposure were higher among respondents covered by a 100 % smoke-free law compared to those not covered by such a law. These findings underscore the importance of efforts to further increase public awareness of the health effects of SHS through education campaigns, which can contribute to changes in social norms regarding the acceptability of smoking around others. Efforts to educate the public about the dangers of SHS are especially important among tobacco users, particularly combustible only users and users of both combustible and noncombustible tobacco. Expanding comprehensive smoke-free policies could further protect all Americans from SHS, a completely preventable health hazard.
